# Metastasis of Breast Cancer Promoted by Circadian Rhythm Disruption due to Light/Dark Shift and its Prevention by Dietary Quercetin in Mice

**DOI:** 10.5334/jcr.203

**Published:** 2021-02-18

**Authors:** Minoru Numata, Akane Hirano, Yukika Yamamoto, Michiko Yasuda, Nobuhiko Miura, Kazutoshi Sayama, Masa-Aki Shibata, Tomohiro Asai, Naoto Oku, Noriyuki Miyoshi, Kayoko Shimoi

**Affiliations:** 1 Graduate School of Integrated Pharmaceutical and Nutritional Sciences, University of Shizuoka, 52–1 Yada, Suruga-ku, Shizuoka, 422–8526, Japan; 2 School of Food and Nutritional Sciences, University of Shizuoka, 52–1 Yada, Suruga-ku, Shizuoka, 422–8526, Japan; 3 Department of Human Nutrition, School of Life studies, Sugiyama Jogakuen University, 17–3, Motomachi, Hosigaoka, Chikusa-ku, Nagoya, Aichi, 464–8662, Japan; 4 Department of Health Science, Yokohama University of Pharmacy, 601 Matano-cho, Totsuka-ku, Yokohama, Kanagawa, 245–0066, Japan; 5 Applied Life Science Course, College of Agriculture, Academic Institute, Shizuoka University, 836, Ohya, Suruga-ku, 422–8529, Japan; 6 Department of Anatomy and Cell biology, Division of Life Sciences, Osaka Medical College, 2–7, Daigaku-machi, Takatsuki, Osaka, 569–8686, Japan; 7 Faculty of Pharma-Science, Teikyo University, 2-11-1 Kaga Itabashi-ku Tokyo, 173-8605, Japan

**Keywords:** light/dark shift, circadian rhythm, breast cancer, lymph node metastasis, quercetin

## Abstract

Epidemiological studies have indicated that a disturbed circadian rhythm resulting from night-shift work is a potential risk factor for breast cancer. However, the mechanism of increased risk of breast cancer by night-shift work remains unclear, and there have been few in vivo studies conducted to definitively associate the two factors. In this study, BJMC3879Luc2 mouse breast cancer cells were transplanted into BALB/c mice. Mice were maintained under lighting conditions that modeled the two-shift system and were investigated for the effect of light/dark cycle disruption on tumor growth and lymph node metastasis. Circadian dysfunction, which was confirmed by measuring circadian locomotor activities using a nano tag device in our light/dark shift model, did not affect tumor growth. However, a significant increase in the number of lymph nodes with distant metastasis was observed. Neutrophil-to-lymphocyte ratio, which is an adverse prognostic factor of breast cancer and also indicator of inflammation, also increased. It has been demonstrated that a chronic inflammatory response is associated with cancer malignancy and poor prognosis in various cancers. These results suggest that night-shift work may also affect distant metastasis and prognosis. In addition, we investigated whether dietary quercetin has anti-metastatic activity against light/dark shift-induced metastasis. A diet containing 0.3 % quercetin significantly inhibited distant lymph node metastasis, particularly metastasis to the iliac and kidney lymph nodes. Our results contribute to our understandings of the effects of the external light environment on breast cancer metastasis and provide a glimpse into potential protective effects of dietary quercetin on light/dark disturbance-induced metastasis.

## 1. Introduction

The mammalian circadian system controls various physiological functions including body temperature, hormone secretion, blood pressure, and sugar metabolism, and is affected by the master clock [1,2,3,4,5]. The master clock synchronizes the rhythm of peripheral clocks to the time of the external environment (i.e., a 24-hour cycle) through stimulation by the light signal received by the eye [6,7,8]. In other words, the light environment surrounding mammals is an important input signal for controlling circadian rhythm. Clock genes controlling biological rhythms interact with each other, and each has its own circadian rhythm [9]. It has been reported that *BMAL1*, a clock gene, has an antitumor effect on ovarian cancer, colon cancer, and head and neck squamous cell carcinoma [10,11,12]. Another study showed that *PER2* is associated with the development of breast cancer [13]. Night and shift workers are inevitably exposed to light at night. Our group previously reported that nocturnal light exposure disturbed the regular expression rhythms of clock genes such as *PER2, BMAL1*, and *CRY2* [5]. The increase in the incidence and mortality rate of breast cancer in women is a worldwide problem. An epidemiological survey of cancer mortality, morbidity, and survival years, conducted in 195 countries and territories from 1990 to 2015 reported that breast cancer is the most common cancer in women (2.4 million cases) [14]. Another study conducted a follow-up survey of female nurses for 10 years and revealed that night or shift work was associated with an increased risk of breast cancer [15]. Furthermore, other epidemiological studies also demonstrated that the potential risk of breast cancer increased in women engaged in shift work [16,17]. Under these circumstances, shift work that results in circadian disruption was categorized as “Group 2A” because it was determined to be probably carcinogenic in the carcinogenic risk assessment by International Agency for Research on Cancer (IARC) in 2007. However, the mechanism of increased risk of breast cancer by shift work remains unclear, and there have been few *in vivo* studies conducted to definitively associate the two factors.

It is known that the survival rate of breast cancer patients markedly decreases as tumor stage progresses, particularly regarding the occurrence of metastasis [18]. Cancer cell migration from breast tissue to lymph nodes can be an important process in breast cancer progression. It has been reported that noradrenaline (NA) contributes to the acquisition of breast cancer cell invasiveness and escape pathways of cancer cells [19,20], although NA is secreted through the activation of the sympathetic nervous system and has a role in the recruitment of lymphocytes to lymph nodes to enhance the body’s defense functions [21]. In our previous studies, it was shown that light exposure during the dark phase activates the stress response pathways and increases secretion of stress hormones adrenaline and NA [5,22]. Hence, internal desynchronization and stress responses caused by disruption of the light environment may affect breast cancer progression and metastasis.

We report here that we investigated the influence of circadian rhythm disruption by light/dark shifts on breast cancer development, especially metastasis, using BALB/c mice transplanted with BJMC3879Luc2 mouse breast cancer cells. We used a light/dark shift cycle pattern that imitated the rotating two-shift work pattern adopted in several types of workplaces, such as production industries, fire departments, and medical institutions [23,24,25]. To clearly disrupt the circadian rhythms of the mice, we chose a radical shift pattern: 12:12 light:dark inverted every two days. BJMC3879Luc2 cells are estrogen receptor positive breast cancer cells derived from metastatic foci within lymph node and lung, and they show a high metastatic potential to lymph nodes and lung [26]. It has been reported that chronic inflammatory responses are associated with the adverse prognosis of various cancer types, including breast cancer. Above all, neutrophil-to-lymphocyte ratio (NLR) correlates with poor prognosis in breast cancer patients [27,28]. Therefore, we also investigated the effect of light/dark shifts on NLR.

We also investigated the preventive effect of quercetin on breast cancer metastasis promoted by circadian disruption in mice implanted with mouse mammary cancer BJMC3879Luc2 cells. Flavonoids are widely distributed in the plant kingdom and their intake has been reported to be inversely correlated with breast cancer risk [29,30]. Quercetin, a representative flavonoid compound, is found in many plant foods such as onions in the O-glycoside form, and its anticarcinogenic effects are receiving attention due to its strong antioxidant activity [31,32]. We have previously shown that Quercetin-3-O-glucuronide (Q3G), a quercetin metabolite, acts as β_2_-AR antagonist and suppresses invasion of human breast cancer cells in vitro [20]. However, there is no report on the suppressive effect of quercetin on metastasis resulting from disruption of the circadian rhythm in mice. Therefore, we investigated the preventive effect of quercetin on light disruption-induced breast cancer metastasis by feeding quercetin mixed diet to breast cancer transplanted model mice.

## 2. Materials and Methods

### 2.1. Cell Culture

A highly metastatic murine BJMC3879Luc2 mammary adenocarcinoma cell line, which is estrogen receptor-positive and expresses *luc2* gene, was a kind gift from Dr. Masa-Aki Shibata (Osaka Medical College, Osaka, Japan) [33]. The cells were maintained in RPMI-1640 medium (Wako, Osaka, Japan) containing 10% heat-inactivated fetal bovine serum (FBS; Invitrogen, Carlsbad, CA, USA) with 50 U/mL penicillin and 50 U/mL streptomycin (Invitrogen, Carlsbad, CA, USA) at 37 °C in 5% CO_2_ in a humidified cell incubator.

### 2.2. Animals and Experimental Light/Dark Conditions

Female BALB/c mice (7 weeks of age) were purchased from Japan SLC (Shizuoka, Japan). Five mice were kept in each plastic cage (W × D × H = 25.5 × 38.8 × 14.0 cm) on soft wood chip bedding in a controlled environment of 23 ± 1ºC, 60% humidity, and 12:12 h light/dark cycles (light on at 8:00) in the Multi-chamber Animal Housing System (Nippon Medical & Chemical Instrument Co., Osaka, Japan). The bedding in each cage was changed and the food and water were refreshed twice a week. For the lighting in each chamber, a high color-rendering cold cathode lamps having a wavelength near-natural light was installed. The light intensity was measured by installing an illuminometer (T-10, Konica Minolta, Tokyo, Japan) on the floor in the cage and set to 250 lux intensity. All animals were provided with MF diet, Oriental Yeast Co., Tokyo, Japan) and water *ad libitum*. Mice were held for 1-week acclimatization period before test initiation and then subjected to experiments at 8 weeks-old. After acclimatization, the light/dark cycle in each experimental group was set as follows: Control, 12:12 h light/dark cycle; Shift, 12:12 light:dark cycle with inverting every two days, which imitated the rotating two-shift work [34] (***Figure 2A***). Lighting time is denoted as Zeitgeber time (ZT), where ZT0 represents the start of the light period (the light period was from ZT0 to ZT12 and the dark period was from ZT12 to ZT24). During the test period, the general condition and body weight of the animals were observed once a week. All procedures were carried out under protocols approved by Animal Experiments Ethics Committee of the University of Shizuoka (Approval number: 165149, 175172).

### 2.3. Locomotor Activity Recording

Under anesthesia with isoflurane, a momentum measuring device (nano tag^®^, Kissei Comtec Co., Ltd. Nagano, Japan) was subcutaneously implanted into the necks of mice. During operation, the animals were cared for with a thermal pad to prevent decrease body temperature. After an acclimatization period of one week, animals were randomly assigned to either of the two types of light/dark cycles and kept for 4 weeks. Locomotor activity was measured by 3-axis accelerometer inside the device. The recording of the momentum data was aggregated every 5 minutes, and the threshold was set to 170. The recorded locomotor activity data was captured by FeliCa communication, and changes in behavioral rhythms were analyzed with the nanotag/Viewer (Kissei Comtec Co., Ltd. Nagano, Japan).

### 2.4. Breast Cancer Model

After acclimatization, mice were randomly divided into Control group and Shift group. BJMC3879Luc2 tumor cells were implanted into the right fourth mammary fat pad of female BALB/c mice by injection of 0.1 mL PBS containing 5 × 10^6^ cells. Mice were kept for 3, 5, or 8 weeks, either under normal or irregular light/dark cycle conditions. Transplanted tumor size was measured with a caliper once a week, and volume was calculated using the following formula: tumor volume = maximum diameter (mm) × (minimum diameter (mm))^2^ × 0.4 [35]. The endpoint for tumor size defined a maximum diameter of 20 mm. Rate of metastasis to lymph node that located distal to the implantation site was calculated as follows: Rate of metastasis (%) = (Number of metastasis to individual lymph node/Total number of mice in each group) × 100.

### 2.5. Ex Vivo Imaging of Metastatic Sites

Prior examination has shown that the development of peak luciferase activity is strongly influenced by depth of anesthesia and body temperature. The general condition of mice varied in distribution from mild to severe. Therefore, ex vivo imaging was performed to avoid the effects of anesthesia depth and body temperature to luciferase activity. For imaging of tumors and metastatic sites, mice received an intraperitoneal (i.p.) injection of 120 mg/kg D-luciferin (Wako) dissolved in PBS. 7 minutes post-injection, tissue-specific metastasis in lung, kidney lymph nodes, and axillary lymph nodes were tracked with bioluminescence using the Vivo Vision IVIS Lumina imaging system (PerkinElmer, Waltham, MA, USA) by measuring luciferase activity for 50 seconds exposure time. Images were analyzed with Living Image software version 3.0.

### 2.6. Determination of NA in Tumor Tissue

To detect intratumoral NA, breast tumors developed in the mammary fat pad were collected. Tumor tissues were homogenized in 0.01 N HCl (containing: 1% sodium disulfite and 1 mM EDTA) and supernatant was collected. NA was measured using the Noradrenaline Research ELISA kit (ImmuSmol, Talence, France) in accordance with the manufacturer’s protocol.

### 2.7. Counting and Classification of Peripheral White Blood Cells

For counting of peripheral white blood cells (WBCs), blood samples were treated with 10-fold the amount of Tuerk’s solution (Sigma-Aldrich, St. Louis, MO, USA), and the number of WBCs per 1 mm^3^ were calculated by hemocytometer.

To perform WBC classification, heparinized blood was smeared onto a slide glass and quickly dried with cool air using a blow-dryer. Slides were fixed and stained with May-Gruenwald stain solution (Sigma-Aldrich) for 3 min, and then stained using 1:20 May-Gruenwald stain solution diluted with phosphate buffer (pH 6.4, M/15, Sigma-Aldrich) for 3 min. Slides were then stained with Giemsa stain solution (Merck, Darmstadt, Germany) diluted 20 times with phosphate buffer for 15 min. Blood smear specimens were inspected (magnification, × 1,000) using immersion oil. One hundred WBCs were counted and classified into five types (i.e., lymphocyte, monocyte, neutrophil, eosinophil, and basophil). Each WBC count per 1 mm^3^ was calculated using the following formula: presence ratio of each type of WBCs × total WBC count.

### 2.8. Feeding a Quercetin-Mixed Diet in the Breast Cancer Model

BJMC3879Luc2 cells were transplanted into female BALB/c mice as described above. The mice were provided MF diet or 0.3% quercetin-containing MF diet. All food was changed three times a week to reduce the effects of deterioration of quercetin in the MF diet. Mice were maintained for 8 weeks in normal light conditions (Control) or light/dark shift environment (Shift). All mice were divided into the following four groups: MF diet (Control) or 0.3% quercetin-containing MF diet (0.3%Q-Ctrl) in a normal light/dark environment; MF diet (Shift) or 0.3% quercetin-containing MF diet (0.3%Q-Sh) in an irregular light/dark shift environment. The lymph node metastasis inhibition rates in 0.3% Q-Ctrl and 0.3% Q-Sh were calculated by following formula using the frequency of metastasis occurrence in the Control or Shift groups as the baseline data: Inhibition (%) = 1–[(rate of lymph node with metastasis/mouse in 0.3% Q-Ctrl or Q-Sh group)/(rate of lymph node with metastasis/mouse in Control or Shift group)] × 100.

### 2.9. Quantitative RT-PCR

Total RNA was extracted from primary tumor, liver, cardiac atrium and cardiac ventricle using TRIzol Reagent (Life Technologies, Carlsbad, CA, USA), and then cDNA was synthesized using a Prime Script RT reagent kit (Perfect Real Time, Takara Bio Inc., Shiga, Japan). RT-PCR was performed using Power SYBR Green PCR Master Mix (Applied Biosystems, Warrington, UK) with specific primers on a StepOne Real-Time PCR System (Applied Biosystems). All specific primer pairs were modeled using web-based software Primer 3 and synthesized at STAR Oligo (Rikaken Co. Ltd., Nagoya, Japan). For PCR primers for *Per2* were 5’-GGCTTCACCATGCCTGTTGT-3’ (forward) and 5’-GGAGTTATTTCGGAGGCAAGTGT-3’ (reverse), the primers for *Pai-1* were 5’-GGTCAGGATCGAGGTAAACGA-3’ (forward) and 5’-TGCCGAACCACAAAGAGAAA-3’ (reverse), and the primers for *Actb* were 5’-GGCTGTATTCCCCTCCATCG -3’ (forward) and 5’-CCAGTTGGTAACAATGCCATGT-3’ (reverse).

The relative gene expression levels were calculated using the 2^-∆∆^^Ct^ method, and normalized against *Actb* mRNA levels.

### 2.10. Statistical Analysis

All data are indicated as mean ± standard error (S.E.M.). Statistical analyses were performed using Student’s T-tests with Pharmaco Basic Statistics (Three S Japan Co., Ltd., Tokyo, Japan). Differences were taken to be significant at *P* < 0.05.

## 3. Results

### 3.1. Disturbances in Light Cycles Cause Breakdown of Circadian Rhythm and Gene Expression

**Figure 1:**
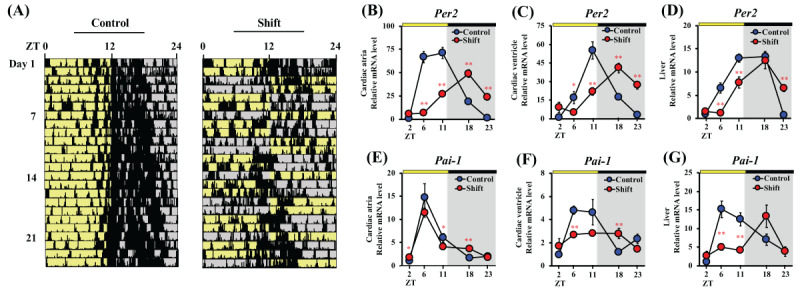
**Circadian locomotor activity profiles, and Per2 and Pai-1 mRNA expression pattern in peripheral tissues.** Circadian activities were measured for approximately 4 weeks using a nano tag. Actograms in the left and right panels represent the Control and Shift groups, respectively (n = 4, A). Locomotor activity levels were measured at 5-min intervals and are indicated by black bars. *Per2* and *Pai-1* expression levels in atria (B and E), ventricle (C and F) and liver (D and G) were analyzed at ZT2, 6, 11, 18, and 23. Values were normalized using *Actb* mRNA levels. Control gene expression levels at ZT2 were defined at as 1. Data represent mean ± S.E.M. (n = 4). *, P < 0.05; **, P < 0.01.

To investigate whether circadian rhythm was impacted by light/dark shifts, a behavior analysis was performed using nano tag^®^ devices. Under regular lighting conditions, active behavior was confirmed from several hours before the lights-out to during the dark period. Moreover, inactive behavior was observed in the light period. In contrast, mice activity during the dark period decreased due to irregular turn-on and/or -off the lights. The peak behavioral time was no longer clear, and some animals seemed to free-run under the shift condition (***Figure 1A*** and S1). Furthermore, the repeated light/dark shift caused dysregulation of expression of the representative circadian clock gene, *Per2* gene in cardiac atrium (***Figure 1B***), cardiac ventricle (***Figure 1C***) and liver (***Figure 1D***), and *Pai-1* gene in cardiac atrium (***Figure 1E***), cardiac ventricle (***Figure 1F***) and liver (***Figure 1G***).

**Figure 2:**
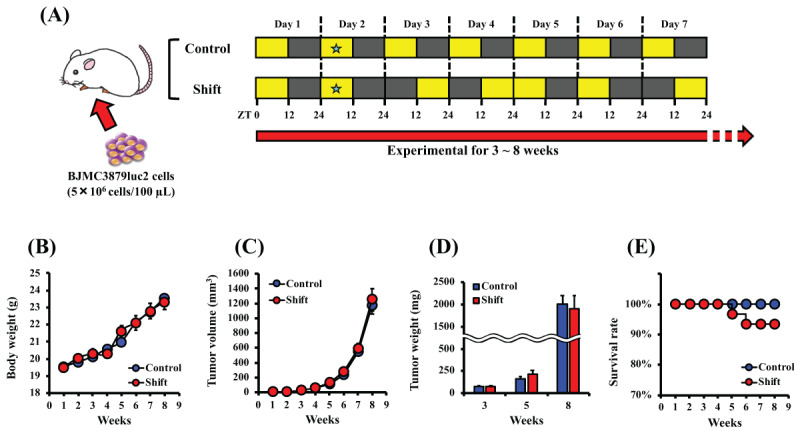
**Disturbance of the light/dark cycle does not affect tumor growth and decrease survival.** Schematic representation of the experimental plan and light/dark conditions (A). Female BALB/c mice were implanted with BJMC3879Luc2 cells in the right fourth mammary fat pad and then maintained under each light/dark cycle condition for 3 to 8 weeks. The lighting conditions of each group were set as follows: Control group, 12:12 h light/dark cycle (lights on at 8:00; ZT0); Shift group, light on ZT0 for 2 days and then conversely on the following 2 days. The star mark (★) indicates the timing of body weight and tumor volume measurement (between ZT7 and ZT8). Mice were killed between ZT7 and ZT8 at 3, 5, or 8 weeks after tumor cell inoculation. Effect of light/dark shifts on body weight (B), tumor volume (C), tumor mass (D), and survival (E). Data represent mean ± S.E.M. (n = 10–30).

### 3.2. Light/Dark Cycle Disruption Promotes Breast Cancer Metastasis

**Figure 3:**
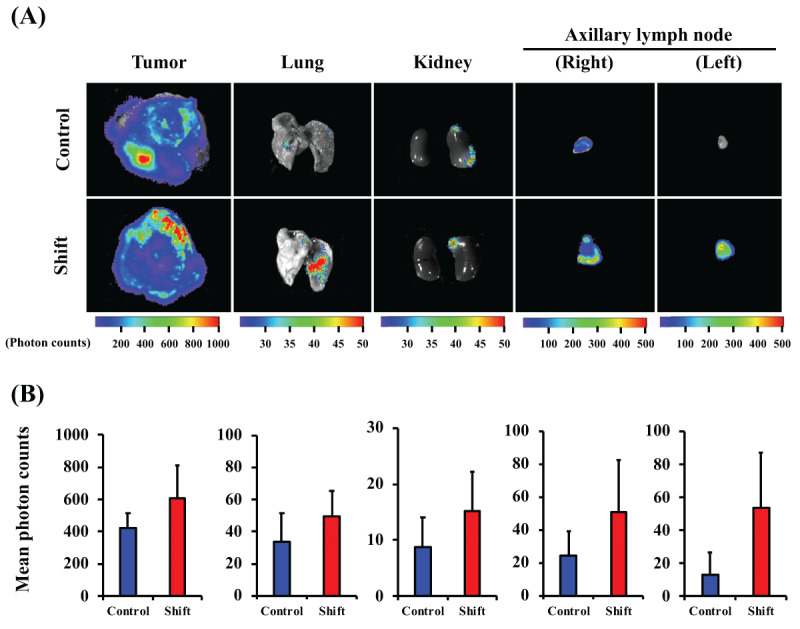
**Bioluminescent imaging of metastatic lesion areas of mice housed under normal or light/dark cycle shift conditions.** Representative bioluminescent images of metastatic lesions obtained at 8 weeks after inoculation (A). Bioluminescent images were obtained 7 min after intraperitoneal administration of luciferin to mice. Quantification of bioluminescent intensities in each tissue taken from mice (B). Regions of interest (ROI) from displayed images were identified in the primary tumor, lung, kidney lymph node, and axillary lymph node, and quantified as photons per second. Data represent mean ± S.E.M. (n = 13–15).

To investigate the effect of irregular light/dark cycles on breast cancer metastasis, BJMC3879Luc2-bearing mice were kept for 3, 5, or 8 weeks under the conditions shown in ***Figure 2A***, and metastases to tissues were evaluated. No difference in body weight (***Figure 2B***), tumor volume (***Figure 2C***), or tumor weight (***Figure 2D***) was observed between normal light conditions and shift conditions. However, in the shift group, the survival rate began to decrease after about 5 weeks of tumor-bearing (***Figure 2E***). In addition, 8 weeks after inoculation, several mice in the shift group had symptoms of crouching, irregular respiration, and piloerection (data not shown). At the terminal sacrifice, cancer cell migration was captured using luciferase activity as a reporter and revealed that luminescence intensity tended to increase in the lung tissue and axillary lymph nodes of the shift group (***Figure 3***).

**Figure 4:**
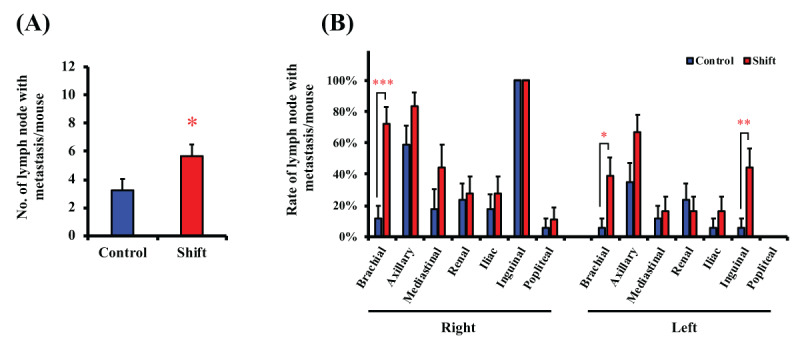
**Frequency of metastasis in lymph nodes.** Number of lymph node metastasis in each group (A) and frequency of metastasis in distant lymph nodes (B). Data represent mean ± S.E.M. (n = 18). *, P < 0.05; **, P < 0.01; ***, P < 0.001.

Necropsy of the major lymph nodes (i.e., axillary, brachial, mediastinal, renal, iliac, inguinal, and popliteal) revealed markedly increased lymph node metastasis per mouse in the shift group (***Figure 4A***). In addition, metastasis in the right inguinal lymph node was observed in all mice (***Figure 4B***). However, the incidence of cancer metastasis in the right brachial lymph node was significantly higher in the shift group (***Figure 4B***). Furthermore, metastasis in the left brachial and inguinal lymph nodes, lymph nodes located distal to the implantation site, were significantly increased by light/dark shifts (***Figure 4B***). These results suggested that disturbances in the light/dark cycle may promote distant metastasis of breast cancer.

### 3.3. Light/Dark Shifts Increase NLR

**Figure 5:**
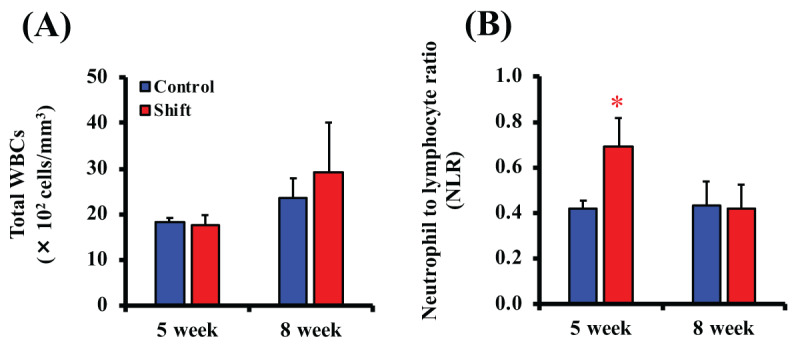
**Total white blood cells (WBCs) and neutrophil-to-lymphocyte ratio (NLR).** Total WBCs were measured at 5- or 8-weeks post-inoculation (A). NLR was calculated from the neutrophil and lymphocyte percentage of WBCs at 5- or 8-weeks post-inoculation (B). Data represent mean ± S.E.M. (n = 6). *, P < 0.05.

The influence of light/dark shifts on the immune system, especially total WBC count and NLR, were analyzed. There was no effect of light/dark shifts on total WBC counts in both groups (***Figure 5A***). However, a decrease in the lymphocyte ratio and an increase in the neutrophil ratio at 5 weeks after cancer cell transplantation were confirmed in the Shift group, and the NLR was significantly higher in the Shift group than in the Control group (1.64-fold, ***Figure 5B***).

### 3.4. NA Content in Primary Tumor

**Figure 6:**
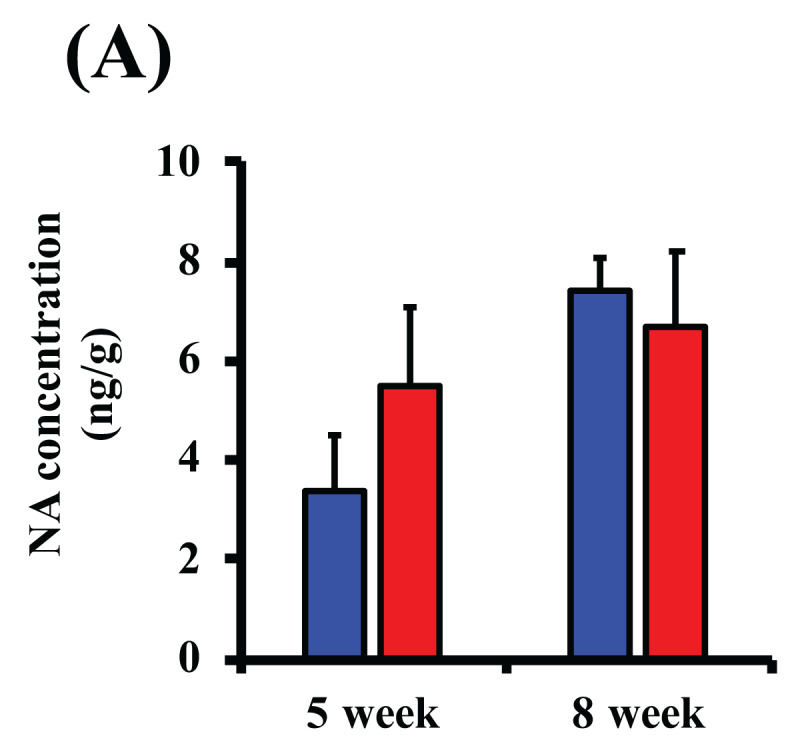
**Change in tumor NA levels.** Intratumoral NA concentration was measured at 5- or 8-weeks post-inoculation. Data represent mean ± S.E.M. (n = 5–10).

Intratumoral NA concentration tended to increase in the Shift group after 5 weeks of inoculation, but the NA concentrations in both groups were not significantly different after 8 weeks of inoculation (***Figure 6***).

### 3.5. Effect of Quercetin on Cancer Metastasis in Vivo

**Figure 7:**
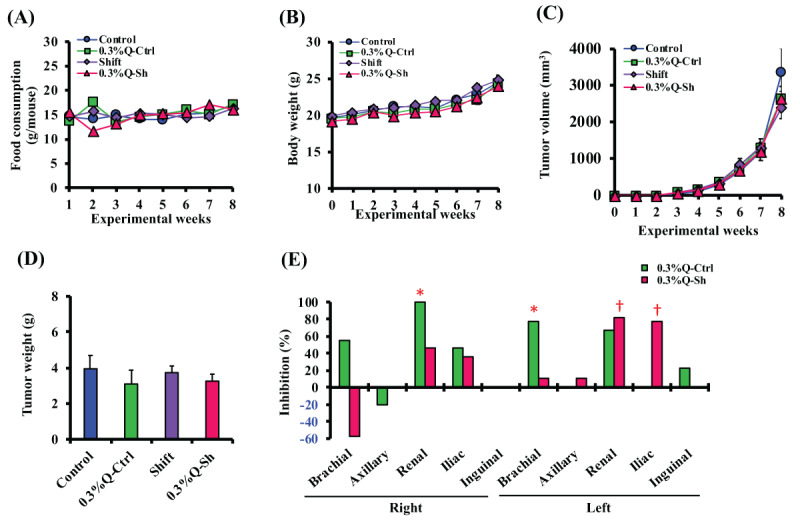
**Effect of 0.3% quercetin diet on the lymph node metastasis.** All animals were given normal MF diet or 0.3% quercetin-containing MF diet over the experimental period. Food intake (A), body weight (B), and tumor volume (C) were measured once a week (n = 6–10). Tumor weight was measured at the final necropsy (D, n = 6–10). Data represent mean ± S.E.M. The lymph node metastasis inhibition rates in 0.3% Q-Ctrl and 0.3% Q-Sh were calculated using the frequency of metastasis occurrence in the Control or Shift groups as the baseline data (E, n = 6–10). *, P < 0.05 vs. control; †, P < 0.05 vs. shift.

To investigate the anti-metastatic effect of quercetin on breast cancer cells, a 0.3% quercetin diet was given to tumor-bearing mice for 8 weeks under the same conditions as described previously (***Figure 2A***). There were no differences in food intake or body weight changes in each group (***Figure 7A*** and ***7B***). There was also no effect of quercetin intake on tumor volume and tumor weight (***Figure 7C*** and ***7D***). Regarding lymph node metastasis, metastasis to various lymph nodes was suppressed in the 0.3%Q-Ctrl and 0.3%Q-Sh groups. In particular, it was observed that metastasis to the kidney lymph nodes and iliac lymph nodes was strongly suppressed (***Figure 7E***).

## 4. Discussion

Breast cancer is the most frequent cancer type in women worldwide. Many previous epidemiological studies reported that shift and night workers are at a higher risk of breast cancer than day shift workers [16,36]. These reports suggest that circadian disruption of biological functions due to deviations from the natural day/night cycle may affect breast cancer development. In this study, the effect of circadian rhythm disruption by light/dark shifts on metastasis of breast cancer was investigated using BALB/c mice transplanted with BJMC3879Luc2 mouse breast cancer cells. A radical shift pattern (12:12 light:dark cycle with inverting every two days) that imitated the rotating two-shift work was chosen to disrupt the circadian rhythms of the mice. The 12-hour rotating shift cycle caused clear circadian disruption and a free-running behavioral rhythm was observed (***Figure 1A*** and Figure S1). Also, the peak times of *Per2* and *Pai-1* were phase advanced about for 7 h (***Figure 1B*** and ***1C***) and 12 h (***Figure 1E*** and ***1F***) in shift group mice, respectively. Furthermore, the expression levels of *Per2* and *Pai-1* showed noticeable downregulation. The association of Per2 or Pai-1 in many types of cancer, including breast, is well-documented [37,38]. Per2 is known to have tumor suppressive activity against mammary tumors, and its down-regulation is advantageous for cancer progression [39]. These outcomes support the hypothesis that light/dark shift accelerate breast cancer progression. Incidentally, the relationship between breast cancer malignancy and Pai-1 has been discussed in several papers and it is presumed that Pai-1 promotes cell migration [40,41].

We demonstrated that disruption of biological rhythms by light/dark shifts promoted tissue metastasis, especially distal lymph node metastasis and further caused a decrease in survival (***Figures 2E, 3*** and ***4***). But it did not affect the tumor volume or weight of transplanted breast tumors (***Figure 2C*** and ***2D***). The effect of biorhythm disturbance on tumor growth and metastasis has been reported in previous study [42], along with downregulation of *per2* by jet lag condition. And, it presents interesting data on the acquisition of the stemness and growth potential of tumor cells and antitumor immunity. In contrast, as mentioned above, our model did not show the effect of light-disturbance on tumor growth. It is difficult to make a general comparison with previous study [42], because of the different tumor model (spontaneous or orthotopic transplant), subtypes of breast cancer cells (estrogen receptor and progesterone receptor sensitivity and HER2 protein expression) and light/dark conditions. Therefore, we think that it will be necessary to consider the systems that more closely resemble human rotating shift work, e.g., comparison with a 3-shift work system (shifted by 8 hours for 3 groups), of different light-dark compositions (6:18 light:dark cycle, and/or 18:6 light:dark cycle) in future studies.

In mammals, various physiological functions including temperature regulation, sleep/arousal, and hormone secretion are known to change periodically over approximately a 24-hour period over a day/night cycle, which is an environmental time signal [1]. Our previous studies revealed that nocturnal light exposure increases secretion of adrenaline and NA through the function of the sympathetic-adrenal-medullary axis (SAM) system and the sympathetic nervous system, the stress response systems, showing evidence that a departure from the natural light/dark cycle is a stress factor [5,22]. A recent investigation demonstrated that chronic stress load activated the sympathetic nervous system accompanied by secretion of NA, and promoted remodeling of tumor lymphatic vessels and cancer metastasis in mice transplanted with MDA-MB-231 cells [43]. We previously reported that NA elevated breast cancer cell infiltration via adrenergic receptors and upregulated MMP9 expression, which contributes to extracellular matrix degradation in MDA-MB-231 cells [20]. These findings suggest that disruption of light/dark cycles causes a stress response accompanied by NA secretion, which may affect breast cancer development. As shown in ***Figure 6***, the concentration of NA in breast tumors tended to increase due to light/dark shifts. However, no increase in *Mmp9* expression in tumors due to light/dark shift was observed (data not shown). As mentioned above, some biological functions including enzyme activity and gene expression show circadian rhythms. It is known that the activity and expression levels of these factors are continuously changing [44,45]. All final sampling in this study was conducted at the same time (ZT 7–8). Therefore, to resolve the relationship between NA and MMP9, further experiments at multiple time points are required in future studies.

We here shown that the NLR is significantly increased by light/dark shifts (***Figure 5B***). At the same time, shortening of survival was observed (***Figure 2E***). It is known that the survival rate of breast cancer patients markedly decreases as tumor stage progresses, particularly regarding the occurrence of metastasis [18]. Cancer cell migration from breast tissue to lymph nodes can be an important process in breast cancer progression. Various internal and external factors affect the metastasis of many cancers including breast cancer through the complex mechanisms, and in recent years there have been many reports that neutrophils have an active role in tumor progression [46,47]. It has been reported that frequent changes in the light/dark environment can induce chronic inflammation and increase the early mortality rate [48]. It has been also reported that the risk of developing liver and lung cancer increases with long-term light/dark shifts [49,50]. In addition, it has been demonstrated that a chronic inflammatory response is associated with cancer malignancy and poor prognosis in various types of cancer [49,51,52]. From these findings, the inflammatory response is an important factor in cancer progression. Peripheral blood NLR is a parameter that reflects systemic inflammation, and it has been shown that a high NLR is associated with poor prognosis in various cancers such as breast, lung, and prostate cancer [28,53]. These outcomes suggest that 12-hour rotating shift cycle is an environmental factor that affects the survival of breast tumor-bearing mice.

To prevent metastasis, treatment with anticancer drugs and/or radiation is performed. On the other hand, adverse effects associated these treatment cause complications and add to increased morbidity and mortality among patients [54]. Therefore, chemoprevention by food is important. We here focused on quercetin contained in plants as a tool to prevent cancer progression (i.e., development, proliferation, and metastasis). Our laboratory previously reported that a quercetin metabolite, Q3G inhibited MDA-MB-231 human breast cancer cell invasion induced by NA via β_2_-adrenoreceptor [20]. It has also been shown that quercetin inhibited the proliferation of MCF-7 human breast cancer cells [55]. In addition, quercetin has an inhibitory effect on cancer cell migration, and it has been reported that cancer metastasis is suppressed by quercetin therapy [56,57,58]. Thus, evidence on the antitumor effect of quercetin is accumulating. However, the suppressive effect of quercetin on metastasis progression as a result of disturbances to the circadian rhythm has not been verified. Therefore, we investigated whether breast cancer metastasis suppression by feeding a 0.3 % quercetin diet is observed or not in breast cancer-transplanted mice under a light/dark shift environment. The effects of quercetin on food consumption and tumor size and weight were not observed in all groups (***Figure 7A, 7C*** and ***7D***). Whereas, metastasis to kidney lymph nodes and iliac lymph nodes was strongly suppressed in mice fed a 0.3 % quercetin diet in a light/dark shift environment (***Figure 7E***). In previous reports of the pharmacokinetics of quercetin and quercetin metabolites, high levels of quercetin and its metabolites were observed in lung, kidney, and liver after quercetin ingestion [59]. Another report showed that Quercetin glucosides administered to the duodenum is partly transported to the iliac lymph vessels [60]. From these studies, it is assumed that the tissue-specific kinetics of quercetin cause local accumulation of quercetin in the iliac lymph nodes and kidney lymph nodes, leading to suppression of cancer cell migration. In examining the preventive effect of the ingestion of quercetin-containing foods on breast cancer, further investigation is required to clarify the detailed mechanism of metastasis inhibition by quercetin.

In conclusion, our study has shown that an irregular light environment that reverses the light and dark phases every two days induced shorter lifespan and high level NLR that is a poor prognostic factor of breast cancer. Circadian rhythm disruption contributes to distal lymph node metastasis even though it did not affect the tumor growth in breast cancer transplanted in mice. These results with breast cancer transplant model and the modified light environment suggest that night-shift work may also affect distant metastasis and prognosis. In addition, in order to investigate the preventive effect of plant components on breast cancer, we fed quercetin mixed diet to breast cancer transplanted model mice and examined the anticancer effect against photodisturbation-induced breast cancer metastasis. We have demonstrated that distant lymph node metastasis due to disruption of the light/dark cycle is suppressed in certain lymph nodes by quercetin. This suggests that quercetin may be useful as a novel tool for the food prevention of light environment-induced breast cancer migration. To the best of we knowledge, this is the first report to the effect of quercetin on distal lymph node metastasis promoted by the disruption of circadian rhythm by the light environment modeled shift worker.

## Additional File

The additional file for this article can be found as follows:

10.5334/jcr.203.s1 Supplementary Figure 1. Circadian locomotor activity profiles of the other mice.

## Abbreviations

DMSO, dimethyl sulfoxide; FBS, fetal bovine serum; IARC, International Agency for Research on Cancer; MMP, matrix metalloproteinase; NA, noradrenaline; NLR, neutrophil-to-lymphocyte ratio; PBS, phosphate buffered saline; Q3G, quercetin-3-o-glucuronide; SAM, sympathetic-adrenal-medullary axis; WBC, white blood cell; ZT, Zeitgeber time.
