# Supplementary material for: Metastasis of Breast Cancer Promoted by Circadian Rhythm Disruption due to Light/Dark Shift and its Prevention by Dietary Quercetin in Mice
Source: J Circadian Rhythms. 2021 Feb 18;19:2. doi: 10.5334/jcr.203 (PMC7894366; doi:10.5334/jcr.203)
Supplement: Supplementary Figure 1. — Circadian locomotor activity profiles of the other mice. [file jcr-19-203-s1.pdf]

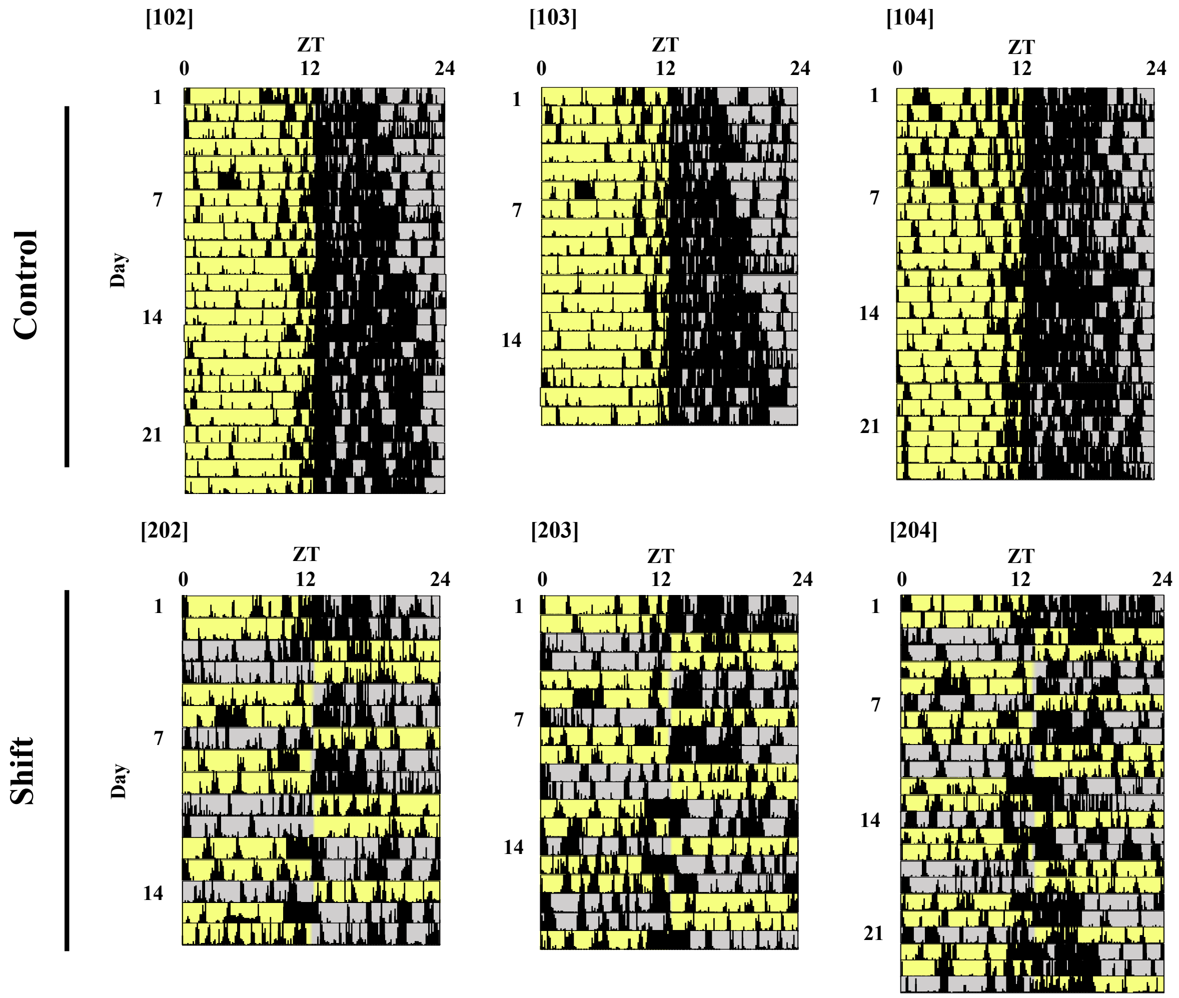

Supplementary figure 1: Circadian locomotor activity profiles of the other mice

Circadian activities were measured for approximately 4 weeks using a nano tag. Individual actograms in the above and below panels represent the Control and Shift groups, respectively. Locomotor activity levels were measured at 5-min intervals and are indicated by black bars. Locomotor activity recording for 103, 202 and 203 are partially lacking due to erroneous operation of a nano-tag.
